# Northerner and Southerner

**Published:** 1890-12-06

**Authors:** 


### Editor's Letter-Box.

[Our Correspondents are reminded that prolixity is a great bar to publication, and that brevity of style and concieness of statement greatly facilitate early insertion.]

### NORTHERNER AND SOUTHERNER.

"An Englishman" writes: In your issue of the 29th ult. there is a review of Mr. Walter Besant's "Captain Cook," where it is remarked, in reference to the hero's parentage, that "from both sides Cook inherited the firmness of purpose, the taciturnity, and self-reliance that mark the north-country folk." If this means anything, it is that the people of the midland and southern counties are deficient in these qualities. I will say nothing as to "taciturnity," but in regard to the rest will your reviewer allow me to ask his authority for drawing a distinction between the north and south of our not illimitable island to the disadvantage of the latter? I am well aware your reviewer has not originated this notion of northern superiority, but what are his reasons for adopting it? History is open to us all, but I see little use in making comparisons, however tempting, and I am well content to subscribe myself "An Englishman."

